# Genetic association analysis of *5‐HTR2A* gene variants in eating disorders in a Mexican population

**DOI:** 10.1002/brb3.1286

**Published:** 2019-06-14

**Authors:** Alma Delia Genis‐Mendoza, David Ruiz‐Ramos, María Lilia López‐Narvaez, Carlos Alfonso Tovilla‐Zárate, Ana Rosa García, Gabriela Cortes Meda, José Jaime Martinez‐Magaña, Thelma Beatriz González‐Castro, Isela Esther Juárez‐Rojop, Humberto Nicolini

**Affiliations:** ^1^ Instituto Nacional de Medicina Genómica (INMEGEN) Ciudad de México México; ^2^ División Académica de Ciencias de la Salud Universidad Juárez Autónoma de Tabasco Villahermosa Tabasco México; ^3^ Hospital General de Yajalón “Manuel Velazco Suarez” Secretaría de Salud Yajalón Chiapas México; ^4^ División Académica Multidisciplinaria de Comalcalco Universidad Juárez Autónoma de Tabasco Comalcalco Tabasco México; ^5^ Hospital Psiquiátrico Infantil Juan N Navarro Secretaría de Salud Ciudad de México México; ^6^ División Académica Multidisciplinaria de Jalpa de Méndez Universidad Juárez Autónoma de Tabasco Jalpa de Méndez Tabasco México

**Keywords:** 5HTR2A gene, Gene, Mexican population, Serotonin

## Abstract

**Introduction:**

The *5‐HTR2A* gene has been implicated as candidate gene for eating disorders. The aim of the present study was to analyze the association of rs6311 and rs6313 polymorphisms of *5‐HTR2A* gene with eating disorders in Mexican population, and to evaluate if the polymorphisms of *5‐HTR2A* gene were associated with comorbidities in eating behavior.

**Methods:**

We conducted a case–control analysis with 460 subjects. We included 168 patients with eating disorders and 292 controls; two polymorphisms of *5‐HTR2A* gene were genotyped. We assessed the association by allele, genotype, and inheritance models. Psychiatric comorbidities were analyzed by genotype in patients with eating disorders.

**Results:**

We found an association between rs6311 and eating disorders in a Mexican population by allele (OR = 8.09; 95% CI = 5.99–11.03; *p* = 2.2e‐16) and genotype (OR = 76.14; 95% CI = 35.61–177.18; *p* = 2.2e‐16). Individuals who carried GG genotype showed increased risk for suicide attempted (OR = 2.14; CI = 1.10–4.26; *p* = 0.035) as comorbidity associated with eating disorders. No positive associations were observed for rs6313 polymorphism.

**Conclusion:**

Our results showed an association of rs6311 (A1438G) polymorphism of *5‐HTR2A* gene with eating disorders, and these polymorphic variants could increase the risk of psychiatric comorbidities. However, more studies are required to replicate the results and to reach to a conclusive association between eating disorders and rs6311.

## 1 INTRODUCTION

Eating disorders are multifactorial and chronic psychiatric conditions associated with aberrant eating behavior and disturbances in body image perception. The most common disorders are anorexia nervosa (AN), bulimia nervosa (BN), and binge eating disorder (BED) (American Psychiatric Association, 2013). The recent literature reports a lifetime prevalence ranging from 0.8% to 3.6% (Mustelin et al., 2016; Stice, Marti, & Rohde, 2013).

The etiology of eating disorders is unknown, however historical studies from family, twin, and adoption studies have suggested a genetic risk on eating disorders (Trace, Baker, Penas‐Lledo, & Bulik, 2013). In this idea, Kaye, Frank, Bailer, and Henry (2005) demonstrated that in cerebrospinal fluid (CSF) of patients with anorexia nervosa, there are lower concentrations of the degradation's products of serotonin (5‐HT) metabolites. Moreover, by Positron‐emission tomography (PET) imaging was found a diminished activity of *5‐HTR2A* on orbital frontal cortex in patients recovered from bulimia nervosa (Kaye et al., 2001). This study suggested that HTR2A gene could be associated with eating disorders.

The *5‐HTR2A* gene is located on the chromosome 13q14‐q21; include three exons and contains > 200 single‐nucleotide polymorphisms (SNPs) along the gene (Gonzalez‐Castro et al., 2013). Two SNPs of *5‐HTR2A* gene have been examined with psychiatric disorders: rs6311 and rs6313. The A1438G (rs6311) polymorphism of *5‐HTR2A* gene is also known as ‐1438G/A. The rs6311 is a nucleotide substitution of guanine for adenine at the position ‐1438 of the promoter region. This variant has been suggested that modulate the *5‐HTR2A* promotor activity in neuropsychiatric disorders. (Parsons, D'Souza, Arranz, Kerwin, & Makoff, 2004) Furthermore, it was associated with eating disorder, although results vary in different populations (Trace et al., 2013). However, the T102C (rs6313) polymorphism of *5‐HTR2A* gene, also known as ‐102C/T, is a substitution of cytosine for thiamine at position ‐102 in the exon 1; this silent variant has been associated in psychiatric disorders as schizophrenia, obsessive‐compulsive disorder (OCD), major depression, and impulsivity in Caucasian and Asian populations (Gonzalez‐Castro et al., 2013; Gray et al., 2018; Kaur et al., 2018; Ni et al., 2013; Sinopoli, Burton, Kronenberg, & Arnold, 2017). Nevertheless, up to the moment there are no association studies of *5‐HTR2A* gene polymorphisms and eating disorders in Mexican population; therefore, we decided to analyze the association of rs6311 and rs6313 polymorphisms of *5‐HTR2A* gene with eating disorders in this population, by (1) a case–control study, and (2) evaluating if the polymorphisms of *5‐HTR2A* gene were associated with comorbidities in eating behavior.

## 2 MATERIALS AND METHODS

### 2.1 Subjects and clinical evaluation

The control group included 292 subjects recruited randomly at the Blood Donor Center in the municipality of Comalcalco, Tabasco; all subjects approved a medical examination prior to blood donation. Trained psychiatrics evaluated via a face‐to‐face interview to ensure a mentally healthy group. As criteria inclusion all the individuals were descendent from Mexican parents and grandparents. The subjects with concomitant illness or neurological disease, and psychiatric antecedents on relatives were excluded. Eighty‐four subjects were included initially. However, to increase the statistical power we selected subjects with age older than 30 years and with a body mass index (BMI) >24 kg/m^2^. These subjects originally belonged to another case–control study previously reported (Lopez‐Narvaez et al., 2015); and the genotypification was previously done along with the original 84 subjects.

For the case group we recruited two samples. The first sample of patients included 94 patients recruited from May 2014 to August 2015. The second sample (*n* = 69) has been recruited from 2016 to date. However, adult patients (*n* = 5) were included from a different case–control study (Saucedo‐Uribe et al., 2019). The patients were evaluated and diagnosed by a medical specialist in psychiatry in the Children's Psychiatric Hospital “Dr. Juan N. Navarro” in Mexico City, Mexico. All the information was gathered in only one psychiatric interview; the diagnoses of eating disorder were based on the DSM‐5 (Diagnostic and Statistical Manual of Mental Disorders, 5th edition). Additionally, the comorbidities associated were established in the case of co‐occurrence with the MINI Kid Spanish version (Mini International Neuropsychiatric Interview for Children and Adolescent). In the same interview, the Questionnaire on Eating and Weight Pattern‐Revised (QEWP‐R) was applied, this in order to identify the characteristics of binge episodes. As criteria inclusion, only Mexican subjects descending from Mexican parents and grandparents were included. Subjects whose missing data were found on the questionnaires; and whose parents or tutors withdrew their consent for participation in the study were excluded. Finally, a total of 168 patients were incorporated in our study.

### 2.2 Ethical statement

All subjects participating in the present study were given verbal and written information related to the research objectives and procedures. The subjects read and signed an informed consent in order to participate in the study; whereas for underage subjects, we ensure that informed consent was given at least for one of his/her parents or tutors. The subjects were informed of anonymity, and not economical remuneration was given from the researchers to require their participation. This study was in accordance with the principles of the Helsinki Declaration in 1975; and approved by Institutional Review Board at Carracci Medical Group at INMEGEN; this study was in compliance with the Code of Ethic of the World Medical Association.

### 2.3 Blood sample collection and genotyping

The blood sample was collected in EDTA tubes for further conservation and storage. Genomic DNA was extracted and purified from peripheral white blood cells following the instructions of Promega's kit (Wizard Genomic DNA Purification). Genotyping of *5‐HTR2A* polymorphism rs6311 and rs6313 for the first sample of patients and control group was determined by polymerase chain reaction end‐point method by means of allelic discrimination following the manufacturer's instructions. For quality control in our genotyping analyses, duplicate samples were genotyped randomly. The polymorphisms (rs6311 and rs6313) studied are available from the TaqMan SNP Genotyping Assay made to order by Applied Biosystems. However, for the second sample of patients (included the adult patients) genotypes of polymorphism rs6311 and rs6313 were extracted from a genetic database constructed with information from the Human Psych Array (Saucedo‐Uribe et al., 2019).

We selected two SNPs of *5‐HTR2A* gene; the rs6311 variant was selected because this polymorphism has been reported associated with patients with anorexia nervosa and bulimia nervosa, although with controversial results (Trace et al., 2013). Polymorphism rs6313 was considered because this variant has been studied and associated with psychiatric pathologies like schizophrenia, major depressive disorder, and obsessive‐compulsive disorder, although there is limited data about the role of rs6313 polymorphism in patients with eating disorders (Ni et al., 2013; Sun, Xu, Zhou, Zuo, & Liu, 2017; Wade, Bulik, Neale, & Kendler, 2000).

### 2.4 Statistical analysis

The clinical characteristics were reported by frequencies and percentages for the categorical variables, and with means and standard deviations for the continuous variables. A χ^2^ test was used to calculate the Hardy–Weinberg equilibrium (HWE); HWE and associations were performed online, freely, and available at https://www.snpstats.net. Odds ratio (OR) with a 95% confidence interval (95% CI) was reported for allelic comparisons and genotype frequencies between cases and controls, as well as comparisons of control group versus AN, BN and BED and comorbidities. The five inheritance models (codominant, dominant, recessive, over‐dominant, and log‐additive) were applied in a logistic regression analysis to estimate the risk of eating disorders between cases and controls. Logistic regression analysis was adjusted by age, gender, and BMI. Statistical analyses were performed with the Statistical Package for the Science Software v.25 (SPSS, Chicago, IL). The level of significance was set at <0.05; and for multiple comparisons the *p*‐value was set at <0.025 using the Bonferroni test. Permutation test was performed in results with statistical differences.

## 3 RESULTS

### 3.1 Clinical characteristics

In the present study, a total of 460 subjects were included in the analysis. The control group include 147 male subjects (50.34%) and 145 female subjects (49.66%). The mean age was 33.97 ± 10.08 years old and the mean BMI was 28.1 ± 4.8 kg/m^2^. For the case group, 168 patients were included, of whom 23.95% were male patients (*n* = 40) and 76.05% were female patients (*n* = 127). Of the 168 cases, 17.86% of the patients had the diagnosis of anorexia nervosa (*n* = 30); 59.52% were patients with bulimia nervosa (*n* = 100); and 22.62% (*n* = 38) were patients with binge eating disorder.

When we analyzed the case group in subgroups (AN, BN, BED), the mean age for the case group was 14.5 ± 3.6 years old (AN [14.2 ± 2.8 years old]; BN [14.8 ± 4.3 years old]; BED [13.8 ± 1.7 years old]) and the mean BMI was 23.9 ± 5.5 kg/m^2^ (AN [18.8 ± 2.9 kg/m^2^]; BN [24 ± 4.7 kg/m^2^]; BED [27.5 ± 6.0 kg/m^2^]). The mean z score for BMI in the subgroups was −0.18 ± 1.17 for AN; 0.87 ± 0.81 for BN and 1.43 ± 0.92 for BED. When we grouped the BMI by diagnosis, we observed 49.69% of patients with normal weight (*n* = 80); the rest of the cases were either in underweight, overweight or obesity (4.35% [*n* = 7]; 18.63% [*n* = 30]; 27.33% [*n* = 44]; respectively).

When the MINI Kid Spanish version was applied, we observed that 90.1% (*n* = 147) of the cases had life time psychiatric comorbidities associated. The most common comorbidities were major depressive episode (*n* = 82), suicidality (*n* = 57), dysthymia disorder (*n* = 36), attention‐deficit/hyperactivity disorder (*n* = 36), generalized anxiety disorder (*n* = 29), oppositional defiant disorder (*n* = 20), and psychotic disorder (*n* = 12).

### 3.2 Comparison of genotypes and allele frequencies

The *5‐HTR2A* polymorphisms rs6311 and rs6313 for the cases and controls satisfied the Hardy–Weinberg equilibrium (rs6311: control group [*p* = 0.21], case group [*p* = 0.7]; rs6313: control group [*p* = 0.16], case group [*p* = 0.7]).

The comparison of distribution of allelic and genotypes between controls and eating disorder is shown in Table 1. We observed an association between polymorphism of *5‐HTR2A* and eating disorders by allele (OR = 8.09; 95% CI = 5.99–11.03; *p* = 2.2e‐16) and by genotype (OR = 76.14; 95% CI = 35.61–177.18; *p* = 2.2e‐16) in the study population. In the analysis by diagnostic groups of eating disorders (AN, BN, BED), we found an association between rs6311 by alleles and genotypes with anorexia nervosa, bulimia nervosa, and binge eating disorder. Table 1. For rs6313 there was a lack of association between this polymorphism and eating disorders in the Mexican population.

**Table 1:** Distribution of allelic and genotypes frequencies of polymorphisms rs6311 and rs6313 in patient with eating disorders

Allele or Genotype	Control (*n* = 292)	Eating Disorders (*n* = 168)	Anorexia Nervosa (*n* = 30)	Bulimia Nervosa (*n* = 100)	Binge eating (*n* = 38)	Statistics							
						Ctrl versus ED		Ctrl versus AN		Ctrl versus BN		Ctrl versus BED	
						OR (95% CI)	*p*	OR (95% CI)	*p*	OR (95% CI)	*p*	OR (95% CI)	*p*
rs6311													
A, *n* (%)	437 (74.83)	90 (26.79)	14 (23.33)	58 (29.00)	18 (23.68)	8.09 (5.99–11.03)	2.2e‐16 9.999e‐05 a	9.65 (5.28–18.76)	3.843e‐16 9.999e‐05 a	7.24 (5.09–10.43)	2.2e‐16 9.999e‐05 a	9.49 (5.51–17.09)	2.2e‐16 9.999e‐05 a
G, *n* (%)	147 (25.17)	246 (73.21)	46 (76.67)	142 (71.00)	58 (76.32)								
A/A, *n* (%)	159 (54.45)	13 (7.74)	2 (6.67)	8 (8.00)	3 (7.89)	76.14 (35.61–177.18)	2.2e‐16 9.999e‐05 a	91.28 (23.10–663.03)	4.076e‐15 9.999e‐05 a	67.27 (28.04–182.92)	2.2e‐16 9.999e‐05 a	79.64 (24.00–382.91)	2.2e‐16 9.999e‐05 a
G/A, *n* (%)	119 (40.75)	64 (38.10)	10 (33.33)	42 (42.00)	12 (31.58)								
G/G, *n* (%)	14 (4.79)	91 (54.16)	18 (60.00)	50 (50.00)	23 (60.53)								
rs6313													
C, *n* (%)	440 (75.34)	246 (73.21)	46 (76.67)	142 (71.00)	58 (76.32)	1.12 (0.82–1.51)	0.525	0.93 (0.48–1.71)	0.944	1.24 (0.87–1.78)	0.264	0.95 (0.53–1.64)	0.965
T, *n* (%)	144 (25.66)	90 (26.79)	14 (23.33)	58 (29.00)	18 (23.68)								
C/C, *n* (%)	161 (55.14)	91 (54.16)	18 (60.00)	50 (50.00)	23 (60.53)	1.76 (0.77–4.04)	0.332	1.45 (0.19–5.95)	0.638	1.99 (0.74–5.05)	0.339	1.66 (0.34–5.76)	0.386
C/T, *n* (%)	118 (40.41)	64 (38.10)	10 (33.33)	42 (42.00)	12 (31.58)								
T/T, *n* (%)	13 (4.45)	13 (7.74)	2 (6.67)	8 (8.00)	3 (7.89)								

Note

Bold indicates statistical significance *p* < 0.025.

ED, eating disorders; AN, anorexia nervosa; BN, bulimia nervosa; BED, binge eating disorder; Ctrl, control group; OR, odds ratio.

a Indicates *p*‐values after 10,000 permutations.

### 3.3 Univariate logistic regression analysis of relationship between each SNP and eating disorder for five inherited models

Table 2 shows the inheritance model analysis of *5‐HTR2A* polymorphisms rs6311 and rs6313 and eating disorders. The genotype G/G was significantly associated with eating disorders in the codominant (OR = 27.19; 95% CI = 7.51–98.46; *p* = 2.2e‐16), recessive (OR = 9.36; CI = 3.12–27.99; *p* = 2.2e‐16), and dominant (OR = 10.66; CI = 3.96–28.68; *p* = 2.2e‐16) models. Likewise, in the additive model (OR = 5.26; CI = 2.77–10.01; *p* = 2.2e‐16) the G allele was demonstrated to be associated with eating disorders. For the polymorphisms rs6313 we found a lack of association between this polymorphism and eating disorders in a Mexican population.

**Table 2:** Analysis of *5‐HTR2A* polymorphisms rs6311 and rs6313 as a function of the inheritance model in patients with eating disorders and controls

Inheritance model	Genotype	Control	Case	Statistic	
				OR (95% CI)	*p*
rs6311					
Codominant	A/A	159 (54.5%)	12 (7.5%)	1.00	2.23e‐16 9.999e‐05^a^
	G/A	119 (40.8%)	61 (37.9%)	5.77 (2.03–16.43)	
	G/G	14 (4.8%)	88 (54.7%)	27.19 (7.51–98.46)	
Dominant	A/A	159 (54.5%)	12 (7.5%)	1.00	2.23e‐16 9.999e‐05^a^
	G/A‐G/G	133 (45.5%)	149 (92.5%)	10.66 (3.96–28.68)	
Recessive	A/A‐G/A	278 (95.2%)	73 (43.5%)	1.00	2.23e‐16 9.999e‐05 a
	G/G	14 (4.8%)	88 (54.7%)	9.36 (3.13–27.99)	
Over dominant	A/A‐G/G	173 (59.2%)	100 (62.1%)	1.00	0.619
	G/A	119 (40.8%)	61 (37.9%)	1.12 (0.49–2.57)	
Log‐addictive	—	—	—	5.26 (2.77–10.01)	2.2e‐16 9.999e‐05 a
rs6313					
Codominant	C/C	161 (55.1%)	88 (54.7%)	1.00	0.394
	C/T	118 (40.4%)	61 (37.9%)	1.07 (0.44–2.52)	
	T/T	13 (4.5%)	12 (7.5%)	0.69 (0.17–2.76)	
Dominant	C/C	161 (55.1%)	88 (54.7%)	1.00	0.922
	C/T‐T/T	131 (44.9%)	73 (45.3%)	0.97 (0.44–2.17)	
Recessive	C/C‐C/T	279 (95.2%)	149 (92.5%)	1.00	0.261
	T/T	13 (4.5%)	12 (7.5%)	0.67 (0.17–2.58)	
Over dominant	C/C‐T/T	174 (59.6%)	100 (62.1%)	1.00	0.670
	C/T	118 (40.4%)	61 (37.9%)	1.13 (0.49–2.59)	
Log‐addictive	—	—	—	0.91 (0.50–1.67)	0.525

Note

Logistic regression adjusted by age, gender, and BMI; Bold indicates statistical significance.

a Indicates *p*‐values after 10,000 permutations.

### 3.4 Analysis of comorbidities by genotypes

Then, we evaluated the association of rs6311 and comorbidities in eating disorders using the recessive model of inheritance. We found an association of G/G genotype of rs6311 in patients with suicide risk (OR = 2.14; CI = 1.10–4.26; *p* = 0.035). The analysis of psychiatric comorbidities among patients with eating disorders and polymorphisms rs6311 is shown in Table 3.

**Table 3:** Comparison of psychiatric comorbidities among patients with eating disorders and *5‐HTR2A* polymorphisms rs6311 using the recessive model of inheritance

Psychiatric comorbidities	Recessive model	Patients without comorbidity n (%)	Patients with comorbidity n (%)	Statistical	
				OR (95% CI)	*p*
Major depressive episode	A/A + G/A	40 (49.38)	34 (41.46)	1.37 (0.74–2.57)	0.391
	G/G	41 (50.62)	48 (58.54)		
Dysthymia disorder	A/A + G/A	57 (44.88)	17 (47.22)	0.91 (0.43–1.93)	0.953
	G/G	70 (55.12)	19 (52.78)		
Major depressive episode + dysthymia disorder	A/A + G/A	29 (50.00)	45 (42.88)	1.33 (0.70–2.55)	0.476
	G/G	29 (50.00)	60 (57.14)		
Attention‐deficit/ hyperactivity disorder	A/A + G/A	58 (45.67)	16 (44.44)	1.04 (0.50–2.24)	0.896
	G/G	69 (54.33)	20 (55.56)		
Suicide risk	A/A + G/A	55 (51.89)	19 (33.33)	2.14 (1.10–4.26)	**0.035 (0.032)** a
	G/G	51 (48.11)	38 (66.67)		
Oppositional defiant disorder	A/A + G/A	66 (46.15)	8 (40.00)	1.27 (0.49–3.48)	0.781
	G/G	77 (53.85)	12 (60.00)		
Generalized anxiety disorder	A/A + G/A	59 (44.03)	15 (51.72)	0.74 (0.32–1.66)	0.583
	G/G	75 (55.97)	14 (48.28)		
Psychotic disorder	A/A + G/A	70 (46.36)	4 (33.33)	1.69 (0.50–6.81)	0.549
	G/G	81 (53.64)	8 (66.67)		

Note

Bold indicates statistical significance.

a Indicates *p*‐values after 10,000 permutations.

## 4 DISCUSSION

We analyzed the association of polymorphism rs6311 and rs6313 of *5‐HTR2A* gene with eating disorders in a sample of Mexican patients and comorbidities according to genotypes in a case–control study in eating behavior.

First, we observed an association between rs6311 and eating disorder in our population in study. Similar to our results, Nishiguchi et al. (2001) found association of allele G in patients with eating disorders in Japanese population. This result was supported in a Canadian population (Bruce et al., 2005; Steiger et al., 2008). In contrast to our result, in Italian population, Ricca et al. (2002) evaluated obese patients with/without BED and reported a lack of association of rs6311 polymorphism with BED. Furthermore, authors reported a lack of association of eating disorder in American, Caucasian, Polish, French, and Japanese population (Ando et al., 2001; Enoch et al., 1998; Fuentes et al., 2004; Kipman et al., 2002; Nacmias et al., 1999; Rybakowski et al., 2003, 2006; Ziegler et al., 1999). Also, other studies reported that the allele associated to eating disorders is the A‐allele; and the population studied with association of A‐allele include Japanese, German, and British population (Campbell, Sundaramurthy, Markham, & Pieri, 1998; Gorwood et al., 2002; Hinney, Ziegler, Nothen, Remschmidt, & Hebebrand, 1997; Kang et al., 2017; Ricca et al., 2004). The discrepancy in our results could be adjudicated to the ethnical race. Other possible cause of discrepancy in our study could be the small size sample included in the studies, possibly overlapping another positive association. In the reports, the range of sample in the reports goes from 75 to 316 patients. Then more studies are necessary but increasing the size of sample. Also, it is necessary that the studies evaluate according to the diagnostic. As in our study, we observed in our Mexican sample that G‐allele carriers of rs6311 have at least ninefold times risk of anorexia nervosa and binge eating, and sevenfold times risk of bulimia nervosa.

Second, we found a significate association of lifetime comorbidities (suicide risk) with rs6311 polymorphism. Similar to our result, Nishiguchi et al. (2001) reported borderline personality disorder (BPD) as comorbidity in patients with anorexia nervosa and bulimia nervosa. The G‐allele (rs6311) was found associated with BPD in Japanese population. Despite the little information on comorbidities in patients with eating disorder, the literature demonstrated that patients with binge eating have high levels of impulsivity (Racine, Culbert, Larson, & Klump, 2009). In this sense, altered behavior like perfectionism or obsessive‐compulsive behavior are traits that persist even after recovery (Gorwood, Kipman, & Foulon, 2003). These findings supported the hypothesis that behavioral traits could be expressed before the onset of the disease. However, genetic variants on specific brain regions as cortical and limbic structures (Kaye et al., 2005), represent a genetic risk to the phenotypic traits in patients with eating disorders. Other hypothesis that could explain the possible association of rs6311 and psychiatric comorbidities in patients with eating disorders, could be a share genetic factor. Although psychiatric disorders are polygenetic, patients with comorbidities, like suicide risk, major depression episode (Wade, Fairweather‐Schmidt, Zhu, & Martin, 2015), and obsessive‐compulsive disorder (Enoch et al., 1998), were analyzed in twin studies, and the presence of a common share genetic factor was suggested as risk factor to other psychiatric disorders; nevertheless, more studies are required in order to enrich the knowledge of possible genetic interaction among associated comorbidities in patients with eating disorders (Anttila et al., 2018).

However, it has been proposed that comorbidity lies on the dysregulation of mood; it could lead to a chronic progression of a full/sub‐threshold bipolar disorder or psychotic disorders on early adulthood (Skjelstad, Malt, & Holte, 2010). Our result demonstrated that the carriers of G‐allele (rs6311) have a double risk to develop a suicide behavior. The knowledge of dual psychiatric alterations in Mexican population could explain the existence of common traits in patients with eating disorders and patients with suicide risk; in this way, non‐established endophenotypes in eating disorders could be associated with carriers of rs6311. However, the treatment of any psychiatric disorder is known to be multimodal (therapy, drugs), and the presence of two or more comorbidities make even difficult to control the symptoms in both disorders; however, genetic biomarkers in these patients could be useful in further association studies to hypothesize the inner relation of psychiatric disorders and to design new therapeutic target in these patients.

At last, we recognized some limitations in our study. The small sample of patients recruited, although it could be attributable to the low collaboration on research of the patients with ED in Mexican population. Furthermore, studies with a large Mexican population are needed in order to replicate the positive association between *5‐HTR2A* polymorphism and eating disorders. We recognized that we only evaluated two *5‐HTR2A* gene polymorphisms of serotonin among the vast existence of polymorphisms related to clinical manifestations on patients with eating disorders. Furthermore, rs6311 and rs6313 are located in a region with high polymorphic variants and we did not perform a haplotype analysis, which could explain if a risk haplotype between the non‐coding rs6311 and rs6313 conferees a risk for eating disorders; moreover, further studies like polygenetic risk score in GWAS or epigenetic studies are needed in order elucidate the role of neurotransmitters genes involved on eating disorders. We acknowledge that only patients with AN, BN, and BED were included in the analysis; and other diagnoses within the broad of eating behavior (pica and rumination) and endophenotypes (compulsivity and impulsivity) were not assessed. We recognized that the number of cases of each eating disorder is not homogeneous and the positive association between rs6311 polymorphism and eating disorders could be overestimated.

In conclusion, our study found that rs6311 polymorphism of *5‐HTR2A* gene is associated with eating disorders in Mexican population. Suicide risk as comorbidity was associated with rs6311 in patients with eating disorders. However, more studies like GWAS and epigenetic studies, with larger samples are needed to gain a deep insight comprehension into the association of *5‐HTR2A* polymorphisms and eating disorders.
